# Supplementary figures and images for: Rapid sex-specific adaptation to high temperature in Drosophila
Source: eLife. 2020 Feb 21;9:e53237. doi: 10.7554/eLife.53237 (PMC7034977; doi:10.7554/eLife.53237)

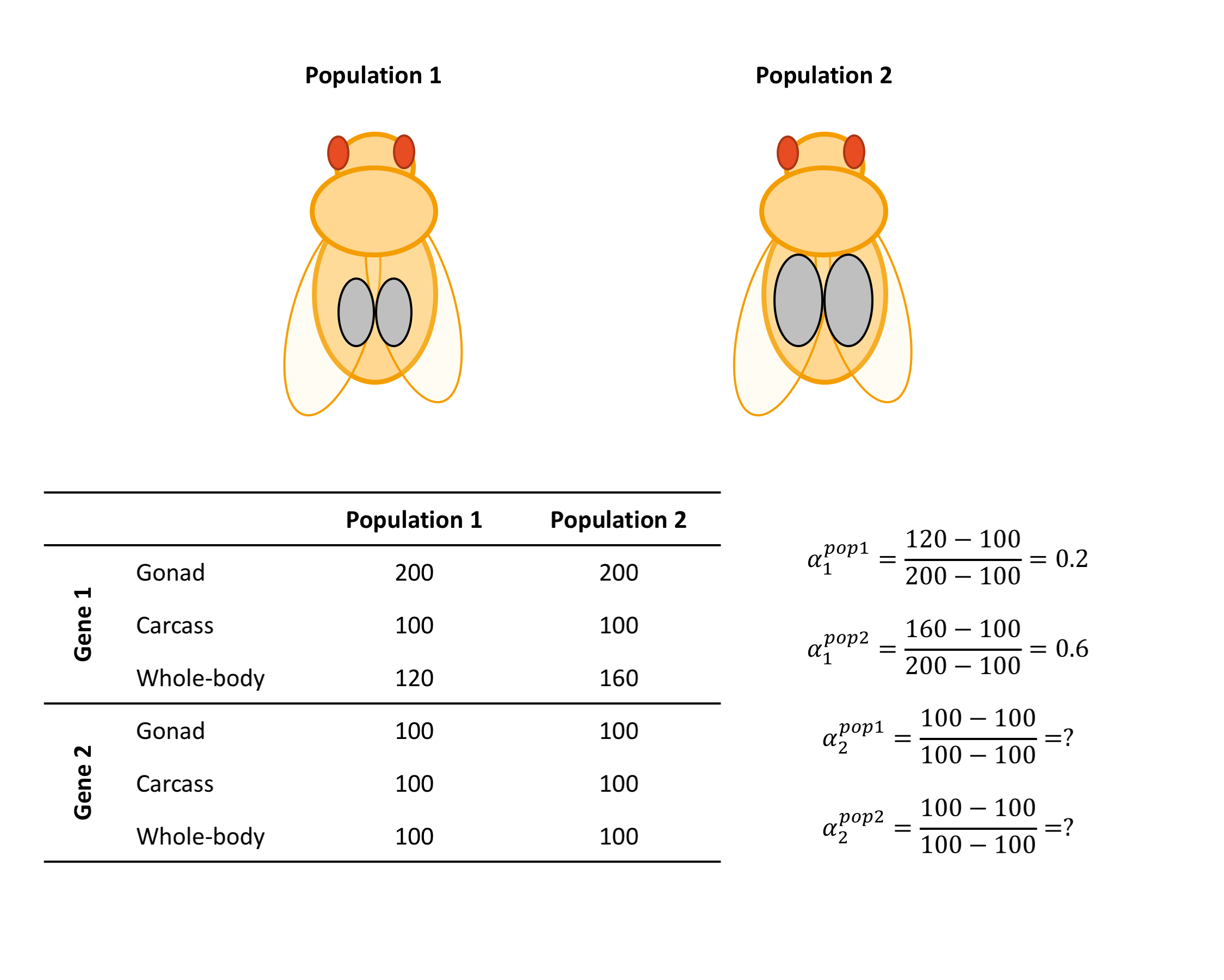

Supplement: Supplementary file 10. — An allometric estimate (αi) measures the abundance of a gene in gonads relative to the overall (mean) abundance in the whole body, reflecting the relative size of gonad in whole body. It may differ between populations. Genes with different expression levels in each tissue (gene1 in the figure) would be affected and thus are informative for the estimation. However, for genes with similar expression in different tissues (gene2 in the figure), they would be affected and the estimation of αi^ would be meaningless. [file elife-53237-supp10.zip › methodFigureS1.png]

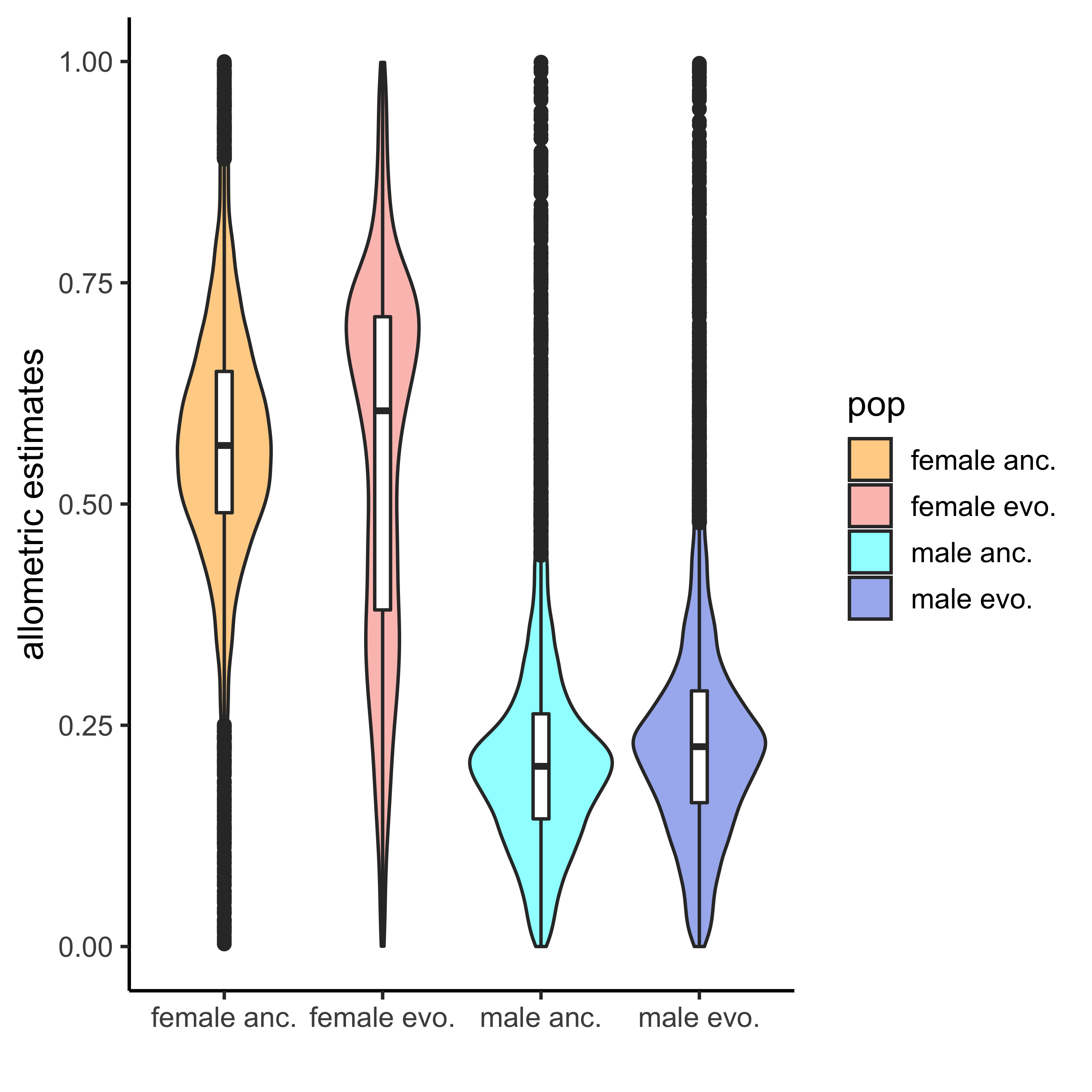

Supplement: Supplementary file 11. — An allmoetric estimate (αi) is the coefficient measuring the abundance of a gene in gonad relative to the overall abundance in the whole body. The distributions of the estimates differ significantly between evolved and ancestral populations in both sexes (Kolmogorov-Smirnov test, D = 0.18 and 0.12 for females and males, respectively; p<0.001 in both tests). [file elife-53237-supp11.zip › methodFigureS2.png]
