# Supplementary material for: Rapid sex-specific adaptation to high temperature in Drosophila
Source: eLife. 2020 Feb 21;9:e53237. doi: 10.7554/eLife.53237 (PMC7034977; doi:10.7554/eLife.53237)
Supplement: Transparent reporting form [file elife-53237-transrepform.docx]

***eLife’s* transparent reporting form**

We encourage authors to provide detailed information *within their submission* to facilitate the interpretation and replication of experiments. Authors can upload supporting documentation to indicate the use of appropriate reporting guidelines for health-related research (see [EQUATOR Network](http://www.equator-network.org/%20)), life science research (see the [BioSharing Information Resource](https://biosharing.org/)), or the [ARRIVE guidelines](http://www.plosbiology.org/article/info:doi/10.1371/journal.pbio.1000412) for reporting work involving animal research. Where applicable, authors should refer to any relevant reporting standards documents in this form.

If you have any questions, please consult our Journal Policies and/or contact us: [editorial@elifesciences.org](mailto:editorial@elifesciences.org).

**Sample-size estimation**

- You should state whether an appropriate sample size was computed when the study was being designed
- You should state the statistical method of sample size computation and any required assumptions
- If no explicit power analysis was used, you should describe how you decided what sample (replicate) size (number) to use

Please outline where this information can be found within the submission (e.g., sections or figure legends), or explain why this information doesn’t apply to your submission:

The sample size of each experiment is stated in the methods section.

- For the two RNA-Seq common garden experiment, the sample sizes can be found in lines 330, 339 and 343.

In summary, 50 flies of each sex from each replicate were freshly frozen or dissected for RNA extraction.

- For the male reproductive activity assay, the sample sizes are given in lines 438-446.

A total of 90 and 60 male flies of the ancestral and evolved populations were assayed for their reproductive activity. (10 for each replicate)

- For the female reproductive dormancy assay, the sample sizes can be found in lines 454-455.

90 female flies from each replicate were dissected for the examination of oogenesis progression.

**Replicates**

- You should report how often each experiment was performed
- You should include a definition of biological versus technical replication
- The data obtained should be provided and sufficient information should be provided to indicate the number of independent biological and/or technical replicates
- If you encountered any outliers, you should describe how these were handled
- Criteria for exclusion/inclusion of data should be clearly stated
- High-throughput sequence data should be uploaded before submission, with a private link for reviewers provided (these are available from both GEO and ArrayExpress)

Please outline where this information can be found within the submission (e.g., sections or figure legends), or explain why this information doesn’t apply to your submission:

The replicate information of each experiment can be found in the same paragraph with sample size in methods section.

- For the experimental evolution, it’s stated in lines 299-300.

10 replicate populations have been independently reared in the novel 18/28°C environment.

- For the two RNA-Seq common garden experiment, the information can be found in lines 316-323 and 339-345.

Main data set:

Five and 30/20 biological replicates for ancestral and evolved populations in males/females.

Correction data set:

Five and 10 biological replicates for each sex and each evolutionary group for whole-body tissue; six biological replicates for each sex and each evolutionary group for carcass and gonadal tissues.

- For the male reproductive activity assay, replicate structure is given in lines 440-446.

In total, 10 biological replicates for each evolutionary group. One and four of the video for the ancestral and evolved populations, respectively, failed the tracking software (flytracker) in more than three attempts and thus were discarded for further analysis.

- For the female reproductive dormancy assay, the sample sizes can be found in lines 454.

Three and 10 biological replicate for each evolutionary group.

- Sequencing reads have been deposited in European Nucleotide Archive (ENA) under the study accession number PRJEB35504 and PRJEB35506

**Statistical reporting**

- Statistical analysis methods should be described and justified
- Raw data should be presented in figures whenever informative to do so (typically when N per group is less than 10)
- For each experiment, you should identify the statistical tests used, exact values of N, definitions of center, methods of multiple test correction, and dispersion and precision measures (e.g., mean, median, SD, SEM, confidence intervals; and, for the major substantive results, a measure of effect size (e.g., Pearson's r, Cohen's d)
- Report exact p-values wherever possible alongside the summary statistics and 95% confidence intervals. These should be reported for all key questions and not only when the p-value is less than 0.05.

Please outline where this information can be found within the submission (e.g., sections or figure legends), or explain why this information doesn’t apply to your submission:

We stated the statistical analysis methods in the corresponding paragraph in the methods section for each analysis we did.

Except for DE analysis where we reported our significance threshold of DE genes at FDR of 0.05, for all other statistical tests, we reported p-value (or FDR) and the corresponding statistics (e.g. odds ratio or W). If p-value (or FDR) is below 0.001, we reported p-value (or FDR) < 0.001. Otherwise, we reported the exact p-values (or FDRs).

(For large datasets, or papers with a very large number of statistical tests, you may upload a single table file with tests, Ns, etc., with reference to sections in the manuscript.)

**Group allocation**

- Indicate how samples were allocated into experimental groups (in the case of clinical studies, please specify allocation to treatment method); if randomization was used, please also state if restricted randomization was applied
- Indicate if masking was used during group allocation, data collection and/or data analysis

Please outline where this information can be found within the submission (e.g., sections or figure legends), or explain why this information doesn’t apply to your submission:

There’re no treatment groups in this study. The fly samples are randomly picked from the reconstituted ancestral or each independently evolved populations.

**Additional data files (“source data”)**

- We encourage you to upload relevant additional data files, such as numerical data that are represented as a graph in a figure, or as a summary table
- Where provided, these should be in the most useful format, and they can be uploaded as “Source data” files linked to a main figure or table
- Include model definition files including the full list of parameters used
- Include code used for data analysis (e.g., R, MatLab)
- Avoid stating that data files are “available upon request”

Please indicate the figures or tables for which source data files have been provided:

Original data for each plot could be found as supplementary files or in the github repository of this study (<https://github.com/ShengKaiHsu/Dsim_sex-specific_adaptation)>, which will be made public upon acceptance.
